# Artificial cell-cell communication as an emerging tool in synthetic biology applications

**DOI:** 10.1186/s13036-015-0011-2

**Published:** 2015-08-12

**Authors:** Stefan Hennig, Gerhard Rödel, Kai Ostermann

**Affiliations:** Institute of Genetics, Technische Universität Dresden, 01062 Dresden, Germany

**Keywords:** Cell-cell communication, Synthetic biology, Cellular consortia, Synthetic circuit engineering, Signal molecule pathways

## Abstract

Cell-cell communication is a widespread phenomenon in nature, ranging from bacterial quorum sensing and fungal pheromone communication to cellular crosstalk in multicellular eukaryotes. These communication modes offer the possibility to control the behavior of an entire community by modifying the performance of individual cells in specific ways. Synthetic biology, i.e., the implementation of artificial functions within biological systems, is a promising approach towards the engineering of sophisticated, autonomous devices based on specifically functionalized cells. With the growing complexity of the functions performed by such systems, both the risk of circuit crosstalk and the metabolic burden resulting from the expression of numerous foreign genes are increasing. Therefore, systems based on a single type of cells are no longer feasible. Synthetic biology approaches with multiple subpopulations of specifically functionalized cells, wired by artificial cell-cell communication systems, provide an attractive and powerful alternative. Here we review recent applications of synthetic cell-cell communication systems with a specific focus on recent advances with fungal hosts.

## Introduction

Cellular communication is a widespread phenomenon in nature, ranging from bacterial quorum sensing [1–4], communication of fungi by pheromones [5, 6] or quorum sensing molecules [7], interactions of microbes with their hosts [8, 9] or with each other [10] to cellular communication in multicellular eukaryotes [11]. These communication systems are either contact-based or rely on diffusible factors. Recently, a further communication mode acting via density pulses in lipid monolayers has been hypothesized [12].

Well characterized communication systems can be adopted into synthetic biology approaches to allow artificial cell-cell communication [13–16]. The design, the engineering and the implementation of artificial circuits and functions within biological systems are exciting fields of research that offer a broad range of applications [17–21]. Synthetic biological circuits have been implemented primarily in prokaryotic cells with *Escherichia coli* (*E. coli*) being the preferred host, but increasingly also in eukaryotic cells and in cellular consortia. A recent review covering a number of excellent studies in the emerging fields of biomedicine and tissue engineering explored the application of synthetic circuits in mammalian host cells [22]. In recent years, several synthetic biology tools – including synthetic riboswitches and ribozymes [23–25] as well as genome engineering tools [26] and a completely synthesized artificial designer chromosome [27] – have also been invented. However, within the fungal kingdom, these were almost exclusively restricted to yeast as a model organism. Despite the enormous importance of fungi in bioprocess engineering and the report of promising approaches for increased yields of biofuels and pharmaceuticals, synthetic biology tools for fungal cells are still in their infancy [28–30].

Bacterial communication systems based on quorum sensing utilize small diffusible molecules (referred to as autoinducers) that are released by bacterial cells and recognized via respective receptors. The extracellular concentration of autoinducers increases with increasing population density. Above a specific threshold, the autoinducer triggers a coordinated density-dependent response within the entire population. Quorum sensing systems have been reported to influence bacterial swarming, the secretion of exoenzymes, biofilm formation and genetic competence [1–4]. In several marine bacteria, bioluminescence is induced at high cell densities via a quorum sensing system, and numerous pathogenic bacteria utilize quorum sensing systems to control the expression of virulence genes [31–33]. Synthetic cellular communication in prokaryotic systems is mainly based on the engineering of bacterial quorum sensing systems [34–36].

Synthetic cellular communication in yeasts and fungi mostly focused on the mating pheromone systems of these organisms [5, 6]. Haploid cells of the commonly used baker’s yeast *Saccharomyces cerevisiae* (*S. cerevisiae*) exist in either of two opposite mating types, referred to as a and α. Both cell types secrete specific small diffusible peptides, acting as pheromones (termed a-factor and α-factor, respectively) and expose receptors for the respective pheromone of the opposite cell type at their surface. Pheromone communication between the haploid cells ultimately leads to zygote formation as part of the yeast’s sexual life cycle. Artificial communication systems in mammalian cells utilize amino acids [37, 38], second messengers [39], growth factors [40], contact-based signaling [41] or volatile compounds [42].

Here, we review recent approaches and applications as well as future challenges of synthetic genetic circuits utilizing artificial communication systems, especially highlighting the advances achieved with fungal host cells. We will focus on different fields of synthetic biology in which the implementation of cellular communication has proven to be beneficial or even mandatory.

### Synthetic quorum sensing systems

**Fig. 1:**
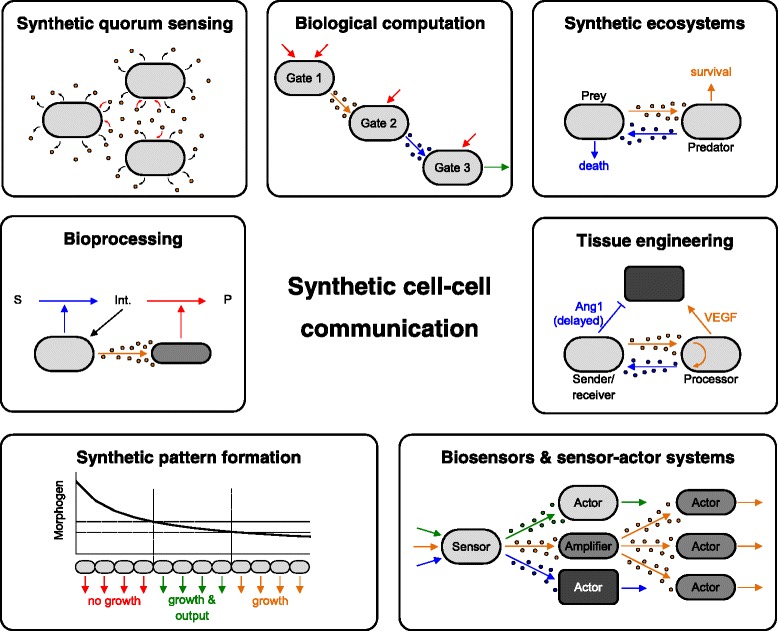
Applications of artificial cell-cell communication. Engineered cellular communication proved to be beneficial or even mandatory in numerous fields of application. These include the implementation of synthetic quorum sensing circuits, biological computation, the design of synthetic ecosystems, bioprocess engineering, biomedicine and tissue engineering as well as the formation of artificial patterns, biosensors and sensor-actor systems. Modified from [38, 78, 87, 133, 148]

In numerous studies, autoinducers have been applied to generate artificial cellular communication in order to synchronize the behavior of an entire population. Rewiring synthetic quorum sensing systems allows the control of target gene expression in a density-dependent manner (Fig. 1), which might be beneficial under certain conditions, e.g., when toxic proteins are produced or excessive protein production leads to a heavy metabolic burden. Such approaches can also circumvent the need for expensive inducer molecules to stimulate the expression of target genes in large-scale fermentations [43]. Rewiring of existing quorum sensing circuits provides the possibility to modify the cellular response to autoinducers (with the final response being graded, threshold-like or bistable [44]), hence synthetic quorum sensing systems may be used to fine-tune the response and to optimize the performance.

Synthetic quorum sensing systems have been implemented in prokaryotic as well as in eukaryotic hosts. By triggering a synthetic quorum sensing circuit with acetate, a common intermediate in *E. coli* metabolism, Bulter et al. achieved the population-density dependent expression of a target gene [45]. Likewise, Chen and Weiss utilized artificial signaling elements to implement cellular communication and quorum sensing behavior in yeast [46]. Sender cells were engineered to secrete isopentenyladenine (IP), a signal molecule implicated in growth and development of *Arabidopsis thaliana*, whereas reporter cells expressed the IP receptor AtCRE1 and its downstream signaling elements. Activation of AtCRE1 in yeast led to the expression of GFP as an output gene from a synthetic promoter (a synthetic response element for SKN7, a nuclear aspartate response regulator of yeast activated by AtCRE1) solely in response to IP secreted by the sender cells. When cells were engineered to secrete IP and respond to it by increased IP synthesis (thus engineering a positive feedback loop), the output gene was expressed in a density-dependent manner. This circuit therefore closely resembled quorum sensing behavior.

Synthetic quorum sensing behavior can also be achieved by rewiring intrinsic intercellular communication systems. Recently, a tunable quorum sensing circuit was reported in yeast by utilizing the α-factor pheromone [43]. First, the pheromone response was coupled to increased pheromone synthesis and GFP expression leading to density-dependent pheromone secretion and fluorescence. Fine-tuning of the circuit’s performance was achieved by engineering of the promoter elements and the pheromone secretion rate. In a second approach, pheromone secretion - and in turn, quorum sensing behavior - was engineered to be inducible upon addition of aromatic amino acids [43]. The resulting circuit responds to the presence of these inducers and the population density, thus implementing a further control element for the desired design. As the yeast *ARO9* promoter employed in this study shows specific response profiles to different aromatic amino acids, the overall circuit’s performance could be fine-tuned by choosing the type and concentration of the aromatic amino acid.

Synthetic quorum sensing behavior has also been implemented in mammalian cells using nitric oxide (NO) as an artificial quorum sensing molecule [39]. Human cells were engineered to synthesize NO, and a positive feedback loop triggered enhanced synthesis of NO upon detection of NO, thus creating a quorum sensing circuit. Fine-tuning was enabled by modifying the NO synthesis rate.

### Biological computation

Biological computation, i.e., the ability of living matter to execute logic functions, has become an emerging issue in synthetic biology [47, 48]. Implementing logic gates within living cells enables them to respond to one or multiple trigger signals or environmental cues in a predefined and predictable manner (Fig. 1). Potential applications range from disease diagnosis, tissue engineering and cellular programming to bioprocessing and biosensing. Theoretically, cell populations or consortia performing logic functions may form autonomous systems that do not require human control, even under varying environmental conditions.

Computation of Boolean functions was achieved in clonal populations of specifically engineered prokaryotic and eukaryotic host cells. Synthetic Boolean gates have been successfully established in *E. coli*, ranging from synthetic AND gates [49–52] to more complex gates with multiple inputs [53] or analog functions [54]. Furthermore, synthetic circuits for counting events [55], push-on push-off switches [56] or oscillators [57], or the detection of the edges of illuminated patterns [58] have been designed. Similarly, logic operations can be performed by clonal populations of yeast [59, 60] or mammalian cells [61–65]. Recent approaches combined biological computation with synthetic memory, based on *in vivo* DNA recombination, thus achieving heritable computation as an exciting step towards the guidance of cellular differentiation in tissue engineering approaches [66, 67].

Performing logic operations in a single population often requires complex and multiple genetic elements to be engineered, which have to be transformed and tested extensively in the desired host. Besides being laborious, this approach may place a heavy metabolic burden on the cells. Distributed computation (logic performed by cellular consortia) might outcompete single-cell logic, especially if highly complex tasks have to be solved [68, 69]. Performing logic operations in microbial consortia allows the individual design and optimization of logic gates distributed throughout multiple subpopulations of the consortium. Physical separation of these gates ensures gate reusability without interference or crosstalk. Due to the reduction in the number of artificial genetic elements to be introduced into a single cell, the metabolic burden decreases, thus leading to enhanced genetic stability, reliability and long-term functionality. Furthermore, cellular consortia utilizing communication systems are able to calculate logic operations in a more robust manner, significantly suppressing misinterpretation of the output resulting from cellular noise [70, 71]. When combining cellular logic with quorum sensing, cells are enabled to calculate the desired function in a density-dependent manner [72–74].

Distributed computation requires the efficient communication of subpopulations in one or more directions. Typically, this is achieved by small diffusible factors (wiring molecules) that are secreted by one type and sensed by a second type of cells, thus allowing one-way communication. Metabolites [75] and bacterial autoinducers [58, 74, 76–78] as well as fungal pheromones [79] have been applied to generate cellular consortia calculating Boolean functions. In alternative approaches, signal transmission from one layer of cells to the next downstream layer was achieved by horizontal DNA transfer using conjugation [80] or bacteriophages [81]. The use of DNA stretches for artificial communication represents a promising strategy as it serves to increase the information capacity that can be forwarded to the receiver cells. Instead of just communicating via signal molecule concentrations and gradients, more complex messages can be passed on to the receivers [80, 81].

In *E. coli* [76, 77] and yeast cells [79] logic gates were implemented in individual populations, and two or more of these populations were wired by diffusible signals. The prokaryotic approaches were based on quorum sensing molecules, while in the eukaryotic system the α-factor pheromones of *S. cerevisiae* and *Candida albicans* were utilized. Employing two different wiring molecules and multiple specifically engineered subpopulations of cells allowed for the implementation of complex logic functions like multiplexers or 1-bit adders with carry in the yeast system. In-depth analysis and quantitative modelling of this approach revealed further potential for fine-tuning and optimization, based on alterations in cell density or by utilizing modified pheromone receptors with reduced affinity [82].

Given the increasing complexity of genetic programs and the need for more complex tasks to be solved, cellular consortia and the implemented communication systems between subpopulations become readily complex, typically associated with increasing noise affecting their reliability. Such systems require a high number of different subpopulations and wiring molecules that are independently secreted and sensed, without interfering with each other. Microfluidic platforms or spatial separation may provide options to use a single wiring molecule for more than one communication channel and thus to limit the required number of different communication systems [68]. Alternatively, distributed output production (i.e., multiple subpopulations are designed to synthesize a similar output in response to individual trigger signals) harbors great potential to efficiently reduce the number of wiring molecules and cellular populations required [69, 83].

### Synthetic ecosystems

Natural ecosystems are intrinsically highly complex, and the vast majority of the interactions between different strains and species have not yet been explored in detail. Analyzing the interactions within such authentic ecosystems is very challenging as neither manipulation of the ecosystem nor in-depth analysis of multiple parameters can readily be achieved within the natural habitats. Synthetic ecosystems, preferably microbial communities, provide a valuable approach to overcome these limitations [84]. Several synthetic interactions between strains and species have been implemented, and some of them closely resemble naturally occurring ecosystems in specific parameters. Given the ease to manipulate synthetic ecosystems (either experimentally or via simulation) it is possible to dissect the major factors leading to a specific ecosystem behavior and to allow for conclusions to be drawn on naturally occurring communities. Although valuable insights were obtained by these synthetic ecologies, they still represent largely simplified communities, and there is still a massive gap to the enormous complexity of naturally occurring microbial ecosystems.

Synthetic circuits for tailored population control have been reported previously. Exploiting a quorum sensing system in *E. coli* by linking the response to the quorum sensing molecule with the expression of a killer gene allowed for a synthetic population control, i.e., predefined steady-state population densities were achieved [85, 86]. By implementing synthetic communication between two populations (prey and predator, Fig. 1), Balagaddé and coworkers established an *E. coli* consortium that exhibited population dynamics similar to authentic ecosystems [87]. Simulations have shown that this system might further be tuned by engineering autoinducer degradation or spatial separation [88, 89]. Rewired quorum sensing systems have also been utilized to control assembly and dispersal of artificial prokaryotic biofilms [90] or to implement obligate symbiosis within an *E. coli* consortium [91].

Simulations performed by Biliouris and coworkers indicated that synthetic obligate mutualism between *E. coli* and yeast might be engineered by utilizing quorum sensing systems [92]. In the respective design, *E. coli* cells secrete a first quorum sensing molecule, which is sensed by the yeast cells and triggers the expression of a resistance gene to detoxify an antibiotic compound. Similarly, yeast cells secrete a second quorum sensing molecule which induces the expression of a resistance gene in *E. coli*, thus leading to the degradation of a second antibiotic. Overall growth of the consortium is only promoted when both species are present in a sufficient density.

Obligate mutualism was also implemented in a synthetic yeast consortium [93]. Two auxotrophic strains, requiring adenine and lysine, respectively, were engineered to secrete high amounts of the supplement needed for growth of the respective other strain. The nutritional cross-feeding established in this study provided insights into mechanisms and population dynamics observed in naturally occurring cooperating communities. In a more recent study, this approach was extended to include a cheater strain requiring lysine secreted by the cooperator strain, but without paying the costs of secreting any common good [94]. Interestingly, some of the co-cultures were dominated by the cooperators, although the cheater strain initially had a higher relative fitness. In these cultures, the cooperators gained a fitness increase due to adaptation to the nutrient-limited environment (adaptive race), which consequently enabled them to dominate the cheaters. This approach provided valuable insights into the mechanisms driving the evolution and maintenance of cooperation in naturally occurring consortia.

Further insight into the population dynamics of cooperators and cheaters within an artificial ecosystem was gained with a yeast consortium in which histidine-requiring cooperator cells secreted invertase (an enzyme that hydrolyses sucrose extracellularly) to grow on sucrose as a single carbon source [95]. The cheater strains did not secrete invertase on their own, but benefitted from the sugars generated by the cooperators’ secreted invertase. Modifying the amount of supplemented histidine allowed for tuning of the costs of the cooperation. Thus, a tunable cooperation within an artificial ecosystem was created, which can contribute to our understanding of the mechanisms facilitating cooperation in natural environments.

Recently, the ability to tune the social behavior of yeast cells was impressively demonstrated [96]: in the experimental study, yeast cells were equipped to secrete and respond to the α-factor pheromone, and by additionally using “sense-only” strains, the authors were able to distinguish between self-communication (asocial behavior) and neighbor communication (social behavior). By tuning parameters such as the cell density, the pheromone secretion rate, expression of *BAR1* (encoding a protease degrading the α-factor) and by implementing positive feedback circuits, the authors were able to achieve versatile ratios of both communication modes, some of which resembled phenomena observed in nature.

Synthetic ecosystems involving communication among different species, which rely on the utilization of volatile acetaldehyde as a common signaling molecule, have also been described [42]. Sender cells from various kingdoms, including prokaryotes, fungi and plants, were engineered to synthesize acetaldehyde that passively diffuses to mammalian receiver cells through the gas phase, thus facilitating artificial one-way communication. Due to the implementation of further interactions, the authors were able to generate synthetic inter-kingdom ecosystems with various behaviors, including commensalism, amensalism, mutualism, parasitism and predation.

### Bioprocess engineering

The conversion of waste products into valuable materials, e.g., for the production of biofuels or biohydrogen, is an emerging field, aiming to increase the independence on fossil fuels. Of special interest is the processing of lignocellulose biomass, a major waste product from agriculture, to generate biofuels. To this end, it is desired to degrade lignocellulose-containing material into soluble sugars and to ferment these sugars to yield biofuels with minimal costs and process times. Exploiting the potential of microorganisms is one of the most promising approaches towards this goal. So far, neither naturally occurring nor genetically engineered organisms proved to perform this task sufficiently. Not only are the costs associated with single culture fermentations still too high to meet industrial demands, it also remains questionable whether such an ideal strain can be engineered at all given the high number of biochemical pathways that need to be tightly controlled for this purpose [30, 97, 98]. Metabolic engineering and the optimization of heterologous expression of numerous enzymes simultaneously might be very time-consuming, and the metabolic burden resulting from the expression of foreign genes may limit further strain optimization. Mixed culture fermentations, including specialists for cellulose degradation and specialists for soluble sugar fermentation, could be the better alternative [99]. Distributing the diverse tasks within a consortium significantly reduces the metabolic burden placed on each single cell type. Individual strains could be optimized in advance and finally integrated into the consortium. Consortia-based approaches allow biochemical reactions with multiple steps to be catalyzed. Compartmentalization of these reactions may reduce the risk of unwanted side-reactions [100]. In comparison to clonal cultures, microbial consortia show enhanced stability against environmental fluctuations or invasion by contaminating species, thus increasing the overall reliability of the process [101]. The use of microbial consortia is expected to enhance product yields by increasing the efficiency of substrate utilization and by removing or converting deleterious byproducts. This strategy may allow obtaining multiple valuable products from a single substrate source [102].

Naturally existing as well as synthetic consortia have been intensively studied in order to convert cellulose biomass into biofuels [99]. Recently, a yeast consortium was engineered for the conversion of cellulose into bioethanol [103, 104]. One strain displayed a scaffold protein at its cell surface, whereas three other strains were engineered to secrete one cellulolytic enzyme each. These enzymes included a specific protein domain which allowed their assembly at the scaffold protein, thus leading to an artificial mini-cellulosome displayed at the surface of yeast cells. By optimizing the ratio of the four strains involved, the authors achieved high ethanol titers (approx. 87 % of the theoretical value). The consortium approach was chosen to reduce the metabolic burden that prevented the engineering of a single strain for scaffold display and enzyme secretion. In a later study, the same authors generated even more complex cellulosomes including several anchor and adapter scaffolds to assemble more cellulolytic enzymes into the protein complex [105]. However, they utilized purified proteins for *ex vivo* assembly as a consortium approach would include numerous strains that have to be co-cultured in a fixed ratio over long time scales.

Synthetic consortia have also been applied to other fields of bioprocessing, including bioremediation, detoxification of byproducts of the chemical industry, food industry and the synthesis of chemicals [106, 107]. In contrast to the intrinsic robustness of naturally occurring microbial communities, synthetic consortia often suffer from a lack of long-term stability that may prevent some of these approaches from entering industrial processes. Several strategies have been proposed to generate stable microbial communities, including the engineering of symbiotic interactions among the consortium members, e.g., by cross-feeding of essential nutrients [93, 94, 108] or by removal of deleterious metabolic waste products [109]. Alternatively, external factors have to be applied, e.g., antibiotics [91, 92] or predefined oxygen tension [110], to maintain a synthetic consortium. Furthermore, spatial [111] or temporal [112, 113] separation as well as embedding of different species into biofilms [114] may provide tools to reach consortium stability. Alternative approaches to control consortia have been reported, including flipping of DNA elements [115], fitness engineering of individual strains [116], or the use of synthetic toggle switches [117–119] that enable cells to switch between two alternative states in response to a trigger signal. Recently, we showed that pheromone-based communication between two yeast species triggers a cell cycle arrest of the pheromone-responding strain, which might provide an option to control the composition of a synthetic consortium over long time scales (see below) [120].

Besides achieving a stable consortium composition, the behavior of individual strains within the microbial community needs to be tightly controlled to ensure maximum yield and reduced process time [99]. With an increasing number of different strains and species included in a synthetic consortium, this task is increasingly challenging. Engineering artificial cellular communication might provide a way to control individual subpopulations within the community to ensure that the sequential action of enzymes (each being expressed by a different strain or species) can be properly timed (Fig. 1). Adapted control of enzyme expression may serve to reduce the metabolic burden and to increase the overall process yield of consortium-based bioprocessing approaches.

In order to achieve an integrated community control by cellular communication, the response of the respective receiver strains to the trigger signals might require further tuning. Using yeast cells as a proof-of-principle, Bashor et al. demonstrated that the response to a pheromone can be modified [121]. Modification of the Mitogen Activated Protein Kinase (MAPK) signaling pathway via scaffold protein engineering allowed for direct coupling of positive or negative regulators to the cascade, thus leading to a modified sensitivity or timing of the cellular response (including accelerated, delayed or pulse-like pheromone response). This opens the way for further options to fine-tune concerted or subsequent events in microbial consortia in a sophisticated manner.

### Biomedicine and tissue engineering

Synthetic biology devices and engineered cells have also been shown to be valuable tools in biomedicine. Engineered prokaryotic cells have been reported which specifically invade cancer cells [122] or detect NO released by inflamed gut tissue [123]. *E. coli* cells were designed to respond to quorum sensing molecules synthesized by *Pseudomonas aeruginosa* (*P. aeruginosa*), a harmful human pathogen [124–126]. In one of these approaches, sensing of the autoinducer induced the synthesis of pyocin S5 and subsequent self-lysis of *E. coli*. The released pyocin S5 acts as a killing agent for *P. aeruginosa* [124]. In another study, engineered *E. coli* cells secreted a novel pathogen-specific toxin upon detecting the autoinducer molecules of *P. aeruginosa* [125]. Furthermore, rewiring the chemotaxis response of the engineered *E. coli* cells enabled them to migrate along the autoinducer gradient towards the pathogen, thus exerting the antimicrobial and biofilm-degrading activities in the close vicinity of the pathogen [126]. These approaches proved to be highly efficient in inhibiting growth and biofilm formation of *P. aeruginosa*, making them attractive tools for future biomedicine applications.

Numerous pathogens utilize quorum sensing systems to initiate the expression of virulence genes or the formation of biofilms [1–4, 31–33]. Interference with the quorum sensing systems of pathogens, e.g., via quorum sensing inhibitors or local excess of quorum sensing molecules, could be a promising strategy to confuse pathogens [2, 3, 31–33, 127, 128]. This approach is particularly interesting because compounds affecting quorum sensing systems typically do not inhibit bacterial growth and thus do not create a high selective pressure. Therefore, the probability to develop resistance against such compounds is rather low. *E. coli* cells secreting quorum sensing molecules of the human pathogen *Vibrio cholera* were shown to significantly reduce the harm of the pathogen’s infection by interfering with the expression of its virulence genes [129].

Most of the potential applications in biomedicine so far rely on engineered *E. coli* cells which are part of the human gut microbiome. Future approaches may also utilize eukaryotic or artificial cells [130, 131] as sensing or actuating entity. Engineering artificial communication between the (bio)medical agent and eukaryotic host tissues has the potential to further improve the success of several therapies, especially for multi-factorial diseases [132].

Tissue development depends on precisely adjusted cellular responses, e.g., the division or differentiation of selected cells. This orchestration requires - in combination with appropriate scaffolds - numerous distinct signal molecules and physicochemical cues to be present at defined time points in a balanced concentration and position. Synthetic biology approaches might provide tools to achieve *in vitro* tissue engineering with the necessary precision and reliability. Recently, a synthetic two-way communication system with two output genes that are expressed with a different timing was applied to guide cellular differentiation *in vitro* [38]. Sender/receiver cells were engineered to synthesize and release tryptophan which was sensed by processor cells, leading to the expression of the Vascular Endothelial Growth Factor (VEGF) as a first output. The response of the processor cells to tryptophan was further coupled to the synthesis and release of acetaldehyde, which was sensed by the sender/receiver cell population and triggered the expression of angiopoietin-1 (Ang1) as a second output in a time-delayed manner. This approach allowed the timing of VEGF and Ang1 synthesis, respectively, eventually inducing transient permeability in vascular endothelial cells, a process that closely resembles blood vessel formation *in vivo* (Fig. 1).

### Synthetic pattern formation

The ability of cells to form patterns is of crucial importance for the development of multicellular eukaryotes. However, the underlying mechanisms that drive these processes are not yet fully understood. The high number of signaling systems (both contact-based and diffusible signals, including formation and maintenance of their gradients), which are active in a developing tissue, is a major hurdle to gain further insights. A valuable approach to simulate these processes in a simplified manner is by engineering synthetic communities that form patterns on their own. The formation of predefined patterns by engineered cells or consortia can help to dissect the mechanisms leading to the development of tissues and to facilitate tissue engineering.

Synthetic patterns have been generated by clonal populations of *E. coli* in response to predefined gradients of antibiotics and inducers. Growth was only possible in a narrow range of concentrations, thus generating a bacterial band-pass filter [133]. Synthetic intercellular communication has also been applied to form predefined patterns of cell growth and reporter gene expression (Fig. 1). By modifying the cellular response to quorum sensing molecules, engineered *E. coli* receiver cells were able to produce a transient output depending on their distance from the autoinducer-releasing sender cells [134] or to build up a “bull’s eye” pattern around the sender cells [135]. Moreover, engineering of *E. coli* cells by the use of synthetic cellular communication resulted in artificial stripe- [136] and ring patterns [137].

Synthetic pattern formation has recently also been shown in a fungal system [138]. Yeast sender cells secreting the α-factor pheromone and responding receiver cells were immobilized in separate compartments within a hydrogel matrix. Diffusion of the low molecular weight peptide pheromone within the matrix enabled cell-cell communication. Expression of the red fluorescent protein (RFP) was used to visualize pheromone response of the receiver cells. RFP expression was dependent on the distance of receiver cells to the compartment boundary and the diffusion time. Tuning was achieved by modifying the density of sender cells and allowed to precisely adjust the area of pheromone responding cells. This approach might be extended to create more complex artificial patterns in hydrogel matrices, e.g., by controlled pheromone degradation or by employing different receiver cells with modified pheromone response profiles [121].

In mammalian cells, synthetic contact-based signaling has been used to propagate a signal along a closed layer of cells. This approach may be utilized for tissue engineering and/or synthetic pattern formation [41]. A mammalian system exploiting a diffusible factor (hepatocyte growth factor, HGF) has been described to yield a synthetic “bull’s eye” pattern in a 3D system [40]. In this study, single cysts of Madin-Darby Canine Kidney cells were locally transfected to secrete HGF (and concomitantly synthesize RFP) in the microenvironment. The response of the receiver cells was coupled to GFP expression, thus yielding a pattern of GFP expressing cells arranged around the HGF sender cells.

### Biosensors and sensor-actor systems

Biological sensor devices are of great importance in numerous fields, including environmental monitoring and human healthcare. Whole-cell sensors, which utilize living cells as a biological recognition unit, represent an emerging branch within the field of biosensors. The sensor entity detects the presence of bioavailable analytes and translates it into a measurable output signal by utilizing analyte-induced or -repressed promoter elements [139, 140]. Besides bacterial whole-cell sensors, yeast-based systems were generated to detect copper ions [141], glucose [142], mycotoxins [143], estrogens [144], organic pollutants in sediments [145] or DNA damaging agents [146]. Devices based on a single sensor cell population might suffer from low signals and/or high background signals due to cellular noise. However, the use of cellular communication may overcome this limitation [70, 71].

In a microfluidic device, Prindle et al. synchronized 2.5 million prokaryotic cells in 500 entities (referred to as biopixels) in a sophisticated manner with a highly constant oscillatory period [147]. Oscillations within a biopixel were synchronized by an engineered quorum sensing system, whereas long-range synchronization of all biopixels was achieved by volatile hydrogen peroxide. By introducing arsenite-dependent regulatory elements in their design, the authors were able to generate a sensor device that tuned its oscillatory amplitude in response to trace amounts of arsenic, thus yielding a biosensor for arsenic contaminations.

Recently, a pheromone-based system for signal amplification in yeast relying on engineered cell-cell communication was reported [148]. Detection of the trigger signal by a population of sender cells was coupled to the secretion of the α-factor pheromone, which in turn induced GFP expression in a population of engineered receiver cells. This consortium approach allowed for signal amplification as compared to the respective device consisting of a single cell type. Further amplification could be achieved by inclusion of a third population of amplifier cells which responded to the presence of the α-factor by increased pheromone secretion, thus leading to higher pheromone concentrations (Fig. 1).

This approach has the potential to engineer autonomous sensor-actor systems that combine sensing of analytes with the expression of target proteins, which in turn result in the processing of the respective analytes. Such systems might be especially interesting for environmental monitoring by coupling the detection of a pollutant with its degradation via the formation of respective enzymes. Utilizing cellular communication would not only amplify the signal, but also reduce the metabolic burden for each cell type and prevent premature expression of the target protein due to cellular noise. Furthermore, by employing different communication molecules, this approach can pave the way to control multiple actor populations by a single sensor cell type, thus generating sensor-actor systems for numerous analytes (Fig. 1). Control of individual subpopulations of actor cells in such a design requires individual targeting, which could be achieved by the use of distinct signal molecules. This might also be a promising approach to include different species in these cellular consortia and to control their behavior individually by the use of proper signals.

**Fig. 2:**
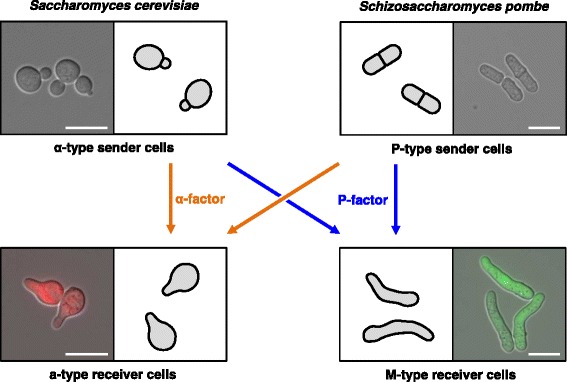
Synthetic inter-species communication in yeast. The yeast species *S. cerevisiae* and *S. pombe* have been designed to communicate artificially via the functional expression and secretion of different pheromones [120, 148]. *S. cerevisiae* cells were engineered to secrete the α-factor pheromone of *S. cerevisiae* or the P-factor pheromone of *S. pombe*, thus providing a possibility for artificial inter-species communication. Likewise, *S. pombe* cells were engineered to secrete either the P-factor or the α-factor pheromone. The pheromone response of the receiver cells of both species is linked to a cell cycle arrest in G1 phase, a characteristic change in morphology (shmoo effect) and to the expression of reporter genes controlled by pheromone-responsive promoters (e.g., the *S. cerevisiae FIG1* promoter controlling the *RFP* reporter gene and *S. pombe sxa2* promoter controlling the *GFP* reporter gene). Microscopic images were captured utilizing an Axio Observer Z1 (Carl Zeiss, Germany). Scale bars represent 10 μm

Yeast cells have been successfully engineered to communicate via heterologous signaling components, e.g., by utilizing the IP-signaling of *Arabidopsis thaliana* (see above) [46]. Gonçalves-Sá and Murray used the pheromone-coding genes and receptors from *Sordaria macrospora* and *Schizophyllum commune* to engineer cellular communication in *S. cerevisiae* [149]. Upon replacement of the native pheromone and receptor genes by the heterologous genes, the yeast cells were able to mate when matching pairs of pheromones and receptors were expressed. Likewise, pheromones and pheromone receptors of different fungal species were previously expressed in yeast to provide insight into fungal pheromone systems [150–153]. Recently, we reported on an inter-species communication system based on the heterologous expression of pheromones [120]. By expressing native or chimeric pheromone-coding genes, *S. cerevisiae* cells were enabled to secrete a pheromone of the fission yeast *Schizosaccharomyces pombe* (*S. pombe*). Likewise, *S. pombe* cells were engineered to secrete the α-factor pheromone of *S. cerevisiae*, thus allowing a two-way communication between both yeast species using their respective pheromones (Fig. 2). Generating further inter-species communication modules, e.g., involving the pheromones of other fungal species, will provide a platform to individually control the behavior of multiple subpopulations in sensor-actor approaches or mixed-species consortia.

## Conclusions

Synthetic biology approaches allow the implementation of numerous devices and systems, within the fields reviewed here and far beyond. With the increasing complexity of the designs and functions to be realized, engineering of the circuits in a single host cell is limited. The rapidly emerging consortium approaches may provide an attractive alternative. They could serve to overcome a number of limitations of synthetic biology tools that act on a single cell level, including intrinsic cellular noise, circuit crosstalk, metabolic burden and genetic instability. It is to be expected that tailored consortia become more and more important for industrial and medical purposes. However, maximum system robustness, product safety and yield can only be assured by a tight control of the systems’ performance.

Transferring designs from the laboratory scale to the conditions required within industrial or medical setups is challenging. The utilization of different strains and growth conditions may lead to deterioration or even failure of synthetic circuits at industrial scales [154], necessitating laborious optimization of the circuits’ components and cultivation conditions. Likewise, the functionality and specificity of synthetic devices for human healthcare need to be assured in order avoid side effects. Communication between the host tissue and the biomedical tool may be used to activate or shut down the implemented synthetic circuits.

Engineering of cellular consortia provides a promising and powerful tool to solve increasingly complex tasks requiring more and more distinct subpopulations to act in a predictable and reliable manner. To engineer cellular communication between the strains and/or species involved in such consortia, a palette of well characterized and standardized modules is indispensable. Although there is a wide spectrum of applications, the number of communication molecules utilized for their realization is still small. Thus, there is a need to integrate novel communication systems into host cells, with minimal crosstalk to their metabolism or to already existing natural and artificial communication systems [100, 155]. We expect new classes of molecules, derived from naturally occurring cell-cell communication systems, to be adopted into artificial cellular communication systems. Quorum sensing inhibitors [9], fungal quorum sensing molecules [7] and fungal pheromones [5] are attractive candidates to open new communication channels within microbial consortia. Evidently, there is a great need to identify the genes required for signal molecule synthesis, signal perception and transduction. Eventually, artificial communication systems may also function across the border of kingdoms, thus allowing for more versatile applications.
